# Neurotransmitter‐Defined Degeneration Patterns in Sporadic and *C9orf72*‐Associated Amyotrophic Lateral Sclerosis: Predilection to GABAergic, Serotonergic, Opioid, Glutamatergic, Endocannabinoid, and Microglial Systems—Implications for Therapy Development

**DOI:** 10.1002/ana.78316

**Published:** 2026-07-21

**Authors:** Marlene Tahedl, We Fong Siah, Jasmin Lope, Jennifer C. Hengeveld, Mark A. Doherty, Russell L. McLaughlin, Orla Hardiman, Ee Ling Tan, Alison Toomey, Jana Kleinerova, Peter Bede

**Affiliations:** ^1^ Computational Neuroimaging Group (CNG), School of Medicine, Trinity College Dublin Dublin Ireland; ^2^ Smurfit Institute of Genetics Trinity College Dublin Dublin Ireland; ^3^ Department of Neurology St James's Hospital Dublin Ireland; ^4^ The Irish FTD/FTLD Network Dublin Ireland

## Abstract

**Objective:**

Amyotrophic lateral sclerosis (ALS) has a markedly distinctive clinical and neuroradiological signature, with the preferential involvement of specific brain networks and the apparent sparing of others. The molecular underpinnings of the strikingly selective anatomical vulnerability have not been fully elucidated to date despite the potential therapeutic relevance of characterizing neurotransmitter receptor‐defined susceptibility to degeneration.

**Methods:**

A large neuroimaging study was undertaken with 258 participants to systematically evaluate topological associations between neurodegeneration and neurotransmitter expression distributions. Patients were stratified based on their genetic profile into sporadic and *C90rf72* hexanucleotide repeat expansion carriers. Anatomical associations were evaluated between patterns of atrophy and topological neurotransmitter receptor distributions. Cross‐sectional and longitudinal trends were comprehensively evaluated over 4 consecutive timepoints.

**Results:**

Our analyses reveal topological associations between neurodegeneration in sporadic ALS and GABA‐A receptor α5‐selective component (GABAa5), serotonin 1a receptor, and kappa opioid receptor expression maps. In addition to these networks, neuronal loss in patients with GGGGCC hexanucleotide repeat expansions exhibit predilection to glutamatergic, endocannabinoid, and microglial systems. Our multi‐timepoint longitudinal analyses reveal dynamic temporal associations between focal volume loss and neurotransmitter expression with increasing spatial associations with noradrenaline transporter and GABAa5, but high attrition rates preclude definite longitudinal inferences.

**Interpretation:**

Our data suggest the preferential vulnerability of GABAergic, serotonergic, kappa opioid mediated networks in sporadic ALS. In *C9orf72*‐assocaited ALS, glutamatergic, endocannabinoid circuits are also susceptible and microglia‐mediated neuroinflammation is also implicated. The comprehensive evaluation of neurotransmitter‐receptor associations not only offer academic insights regarding pathophysiological processes in ALS, but may inform targeted therapy development strategies. ANN NEUROL 2026;100:948–961

Research efforts in amyotrophic lateral sclerosis (ALS) have gained unprecedented momentum in recent years, but there are no effective disease‐modifying therapies in sight for most patients. A small percentage of patient carrying specific genetic variants may be eligible for antisense oligonucleotide (ASO) therapies and enrolment into genotype‐specific pharmaceutical trials. One of the active frontiers of ALS research is the characterization of the presymptomatic phase of the disease, but similar to breakthroughs in ASO therapies, presymptomatic research has solely focused on either *SOD1* or *C9orf72* carriers.^1^ Clinical and neuroimaging studies^2^, ^3^, ^4^ of presymptomatic cohorts have revealed accruing disease burden long before disease manifestation, but these insights are unlikely to be directly transferrable to those not carrying these specific genetic variants. There may be a risk to extrapolate these observations to “sporadic” cohorts, and similarly, there is also a risk of over‐interpreting observations from specific transgenic animal models. The majority of patients with ALS seen in our clinics, have no confirmed family history of ALS and test negative for large panels of ALS‐associated genetic variants. The exact definition of “sporadic” patients varies, but for practical purposes, these are patients who have no known family history of ALS. By the time these patients present to a specialist neuromuscular clinic, they would have typically experienced symptoms for a number of months^5^ and considerable cerebral and spinal neurodegenerative changes have already occurred. These patients account for 90 to 95% of patients with ALS and are not eligible for gene therapies at present. Understanding disease propagation mechanisms in this “non‐familial,” seemingly “gene‐negative” cohort is crucial to develop viable pharmacological strategies for the vast majority of patients affected by ALS.

Clinical, neuroimaging, and neuropathology studies are concordant in demonstrating that patients with ALS exhibit strikingly selective cerebral atrophy with the predilection to specific brain regions and the relative sparing of others.^6^ Phylogenetic and ontogenetic factors have been suggested to explain why certain brain networks may be particularly vulnerable to ALS^2^, ^7^ and why other brain regions remain relatively spared.^8^ Metabolic, mitochondrial, genetic, inflammatory, and glial factors have also been consistently suggested to explain regional brain vulnerability differences.^9^ Disease propagation mechanisms are also relatively poorly understood in ALS, despite the suggestion of prion‐like, trans‐synaptic processes, connectivity‐based spread, and network‐wise degeneration.^10^ Staging‐systems have been proposed based on clinical milestones and pTDP‐43 patterns,^6^ but these do not offer practical targets for drug development. A possible interface of focal metabolism, selective initial disease burden, trans‐synaptic spread, and connectivity‐based network‐wise propagation are physiological neurotransmitter expression and receptor density patterns. Glutamate‐mediated excitotoxicity, impaired GABA‐linked inhibition, and serotonergic dysfunction have long been implicated in ALS,^11^ but topological associations between progressive neurodegeneration and physiological neurotransmitter concentrations have not been systematically evaluated in vivo. Accordingly, a large longitudinal neuroimaging study has been carried out to systematically evaluate a large panel of neurotransmitters and their association with progressive degeneration in a genetically stratified cohort of patients with ALS.

## Methods

### Participants

This research study was approved by the Ethics Committee of Beaumont Hospital, Dublin, Ireland (REC reference: 08/90) and all participants provided written informed consent. A total of 145 patients with ALS and 113 healthy controls (HC) were included in this study and up to 4 longitudinal neuroimaging timepoints were included with a uniform inter‐scan interval of 4 months for a subset of patients. A total of 145 patients scanned at timepoint 1, 72 patients scanned at timepoint 2, 47 patients scanned at timepoint 3, and 24 patients scanned at timepoint 4. Core clinical and demographic variables were systematically recorded for each participant on the day of the scan, including age, sex, symptom‐onset‐to‐scan interval, handedness, years of education, site of onset, family history of ALS or frontotemporal dementia (FTD) in first‐ or second‐degree relatives, and the revised ALS Functional Rating Scale (ALSFRS‐r) score (Table 1). Patients were diagnosed according to the revised El Escorial criteria. Exclusion criteria included prior neurosurgery, cerebrovascular events, traumatic brain injury, or comorbid neoplastic or neuroinflammatory conditions. Patients without genetic data were also excluded. For HCs, exclusion criteria included any neurological, neurosurgical, or psychiatric history as well as a family history of ALS, FTD, Parkinson's disease, Huntington's disease, or Alzheimer's disease. Incidental radiological findings, such as meningiomas, arachnoid cysts, or prior strokes also led to exclusion. Group comparisons of demographic variables (ALS vs HC at baseline) used independent *t* tests for continuous variables (age, education) and χ^2^ tests for categorical variables (sex and handedness). Test statistics, degrees of freedom, and *p*‐values are reported in Table 1. Baseline ALSFRS‐r scores were compared between C9NEG and C9POS using Welch's *t* test. Longitudinal changes in ALSFRS‐R for C9NEG were assessed with paired *t* tests between consecutive sessions and the Friedman test across all 4 sessions, using complete‐case data. Statistics were computed in *RStudio* (version 2022.12.0 + 353; R Version 4.2.2).

**TABLE 1:** Demographic and Clinical Profiles of Study Participants at Baseline, Stratified by Genetic Status.

	C9POS (n= 15)	C9NEG (n = 130)	All ALS (n = 145)	HC (n = 113)	Statistics (all ALS vs HC)
Age, yr (mean ± SD)	55.7 ± 7.2	59.8 ± 11.8	59.4 ± 11.5	59.4 ± 10.7	*t*(256) = 0.022, *p* = 0.983
Sex, M/F	10/5	88/42	98/47	56/57	*χ* ^*2*^(1) = 7.846, *p* = 0.005
Handedness, R/L	13/2	119/11	132/13	106/7	*χ* ^*2*^(1) = 0.349, *p* = 0.554
Education, yr (mean ± SD)	13.3 ± 3.8	13.4 ± 3.5	13.4 ± 3.5	14.7 ± 3.5	*t*(256) = −2.960, *p* = 0.003
Symptom duration, mo (mean ± SD)	16.3 ± 4.4	15.9 ± 5.0	16.0 ± 4.9	N/A	N/A
Total intracranial volume, ml (mean ± SD)	1459.1 ± 95.9	1559.4 ± 169.6	1549.0 ± 166.1	1502.8 ± 154.1	*t*(256) = 2.287, *p* = 0.023
ALSFRS‐R at (mean ± SD)	31.3 ± 11.5	38.9 ± 5.8	38.0 ± 7.2	N/A	*t*(14.8) = 2.526, *p* = 0.023 (C9NEG vs C9POS)

Values are presented as mean ± SD for continuous variables and counts for categorical variables.

ALS = amyotrophic lateral sclerosis; ALSFRS‐r = the revised ALS Functional Rating Scale; C9POS = C9orf72‐positive ALS; C9NEG = sporadic ALS patients without known ALS‐associated genetic variants; F = female; HC = healthy controls; L = left‐handed; M = male; mo = month; N/A = not available; R = right‐handed; SD = standard deviation; yr = years.

### Genetics

ALS patients were stratified by their genetic status: patients carrying GGGGCC hexanucleotide repeat expansions (HRE) in *C9orf72* (“C9POS” n = 15) and sporadic patients with no family history of ALS or FTD testing negative for a panel of ALS‐associated genetic variants as well as *C9orf72* HRE (“C9NEG”, n = 130). Methods for genetic screening have been described previously.^12^ Briefly, each participant has been screened for HRE in *C9orf72* by repeat‐primed polymerase chain reaction (PCR) and a panel of protein‐altering, exonic or splice‐site variants present in 32 genes linked to ALS in the ALS online database (ALSod) including *ALS2*, *ANG*, *ATXN2*, *CHCHD10*, *CHMP2B*, *DAO*, *DCTN1*, *ELP3*, *ERBB4*, *FIG4*, *FUS*, *HNRNPA1*, *MATR3*, *NEFH*, *NEK1*, *OPTN*, *PFN1*, *PRPH*, *SARM1*, *SETX*, *SIGMAR1*, *SOD1*, *SPAST*, *SPG11*, *SQSTM1*, *TAF15*, *TARDBP*, *TBK1*, *UNC13A*, *UBQLN2*, *VAPB*, and *VCP*.

### Neuroimaging

All scans were uniformly acquired on the same 3 T Philips Achieva scanner with the same head coil using a standardized protocol. A high‐resolution T1‐weighted (T1w) 3 dimensional (3D) inversion recovery‐prepared spoiled gradient recalled echo sequence was acquired with pulse repetition time/echo time = 8.5/3.9ms, inversion time = 1,060ms, and field of view = 256 × 256 × 160mm. T1w images were pre‐processed using the Computational Anatomy Toolbox (CAT12)^13^ within SPM12. The pipeline included denoising, bias correction, affine registration, local adaptive segmentation, skull stripping, and spatial normalization to Montreal Neurological Institute (MNI) space via DARTEL and geodesic shooting. Modulated gray matter (GM) concentration maps were then scaled by the Jacobian determinant of the deformation field to preserve local GM volume. Confounding variables such as age, sex, handedness, education, and total intracranial volume (TIV) were regressed out from the GM maps using SPM12 to generate residual GM maps for downstream analyses.

Spatial correlations between residual GM maps and 44 positron emission tomography (PET)‐derived neurotransmitter receptor density maps (Table 2) were examined using the JuSpace toolbox.^14^ The spatial distribution of the following receptors were considered: serotonin 1a receptor (5HT1a), serotonin 1b receptor (5HT1b), serotonin 2a receptor (5HT2a), serotonin 4 receptor (5HT4), serotonin 6 receptor (5HT6), nicotinic α4β2 receptor (A4B2), cannabinoid type 1 receptor (CB1), cerebral blood flow (CBF), cerebral metabolic rate of glucose (CMRglu), cyclooxygenase‐1 (COX1), dopamine D1 receptor (D1), dopamine D2 receptor (D2), dopamine transporter (DAT), fluorodopa (FDOPA), GABA‐A receptor α5‐selective component (GABAa5), GABA‐A receptor benzodiazepine binding site (GABAa), histone deacetylase (HDAC), kappa opioid receptor (KOR), muscarinic acetylcholine receptor M1 (M1), mu opioid receptor (MOR), muscarinic acetylcholine receptor (MU), activated microglia (Microglia), norepinephrine transporter (NAT), NMDA receptor (NMDA), serotonin transporter (SERT), synaptic vesicle glycoprotein 2A (SV2A), vesicular acetylcholine transporter (VAChT), vesicular monoamine transporter 2 (VMAT2), and metabotropic glutamate receptor 5 (mGluR5). Spatial correlation analyses were run with 10,000 permutations. Cross‐sectional comparisons included C9NEG versus HC (n = 113), C9POS versus HC (n = 15), and C9POS versus C9NEG (n = 15) contrasts, with additional regression for symptom duration. We note that JuSpace requires equal sample sizes in both groups for comparisons, so the number of scans used for the statistical comparisons was less than what was available. For longitudinal analyses, only the C9NEG group was considered for power reasons (Table 1). Residual GM maps from sessions 1 to 4 were compared to HC session 1 residuals. Longitudinal trends were assessed by performing linear regression of the mean Fisher's z values against session number in *RStudio*. Statistical significance was evaluated using *p*‐values with Benjamini–Hochberg false discovery rate correction (p_FDR_BH). The JuSpace toolbox generates this output, along with median Spearman's rho and mean Fisher's z values. The results were visualized in *RStudio* and MATLAB (version R2024b, The MathWorks, Natick, MA).

**TABLE 2:** Overview of the 44 PET‐Derived Neurotransmitter Receptor/Transporter Maps used for Spatial Correlations to GM Residual Maps

Map	Full name	Reference	Map	Full name	Reference
5HT1a	Serotonin 1a receptor	Savli et al, 2012 (https://doi.org/10.1016/j.neuroimage.2012.07.001)	HDAC	Histone deacetylase	Wey et al, 2016 (https://doi.org/10.1126/scitranslmed.aaf7551)
5HT1a (map 2)	Serotonin 1a receptor	Beliveau et al, 2017 (https://doi.org/10.1523/JNEUROSCI.2830‐16.2016)	KOR	Kappa opioid receptor	Shokri‐Kojori et al, 2022 (https://doi.org/10.1002/hbm.25733)
5HT1b	Serotonin 1b receptor	Savli et al, 2012 (https://doi.org/10.1016/j.neuroimage.2012.07.001)	KappaOp	Kappa opioid receptor (same as KOR)	Vijay et al, 2018 (https://doi.org/10.1016/10.1016/j.bpsc.2020.10.013)
5HT1b (map 2)	Serotonin 1b receptor	Beliveau et al, 2017 (https://doi.org/10.1523/JNEUROSCI.2830‐16.2016)	M1	Muscarinic acetylcholine receptor M1	Naganawa et al, 2020 (https://doi.org/10.2967/jnumed.120.246967)
5HT2a	Serotonin 2a receptor	Savli et al, 2012 (https://doi.org/10.1016/j.neuroimage.2012.07.001)	MOR	Mu opioid receptor	Shokri‐Kojori et al, 2022 (https://doi.org/10.1002/hbm.25733)
5HT2a (map 2)	Serotonin 2a receptor	Beliveau et al, 2017 (https://doi.org/10.1523/JNEUROSCI.2830‐16.2016)	MOR (map 2)	Mu opioid receptor	Vijay et al, 2018 (https://doi.org/10.1016/10.1016/j.bpsc.2020.10.013)
5HT4	Serotonin 4 receptor	Beliveau et al, 2017 (https://doi.org/10.1523/JNEUROSCI.2830‐16.2016)	MU	Muscarinic acetylcholine receptor	Kantonen et al, 2020 (https://doi.org/10.1016/10.1038/mp.2017.183)
5HT6	Serotonin 6 receptor	Radhakrishnan et al, 2018 (https://doi.org/10.2967/jnumed.117.206516)	MU (map 2)	Muscarinic acetylcholine receptor	Turtonen et al, 2021 (https://doi.org/10.1016/j.bpsc.2020.10.013)
A4B2	Nicotinic α4β2 receptor	Hillmer et al, 2016 (https://doi.org/10.1016/j.neuroimage.2016.07.026)	Microglia	Activated microglia / neuroinflammation	Lois et al, 2018 (https://doi.org/10.1021/acschemneuro.8b00072)
CB1	Cannabinoid type 1 receptor	Laurikainen et al, 2019 (https://doi.org/10.1016/j.neuroimage.2018.10.013)	NAT	Norepinephrine transporter	Hesse et al, 2017 (https://doi.org/10.1007/s00259‐016‐3590‐3)
CB1 (map 2)	Cannabinoid type 1 receptor	Normandin et al, 2015 (https://doi.org/10.1016/j.neuroimage.2018.10.013)	NMDA	NMDA receptor	Galovic et al, 2023 (https://doi.org/10.1001/jamaneurol.2022.4352)
CBF	Cerebral blood flow	Holiga et al, 2018 (https://doi.org/10.1371/journal.pone.0206583)	SERT	Serotonin transporter	Fazio et al, 2016 (https://doi.org/10.1016/j.neuroimage.2016.03.019)
CMRglu	Cerebral metabolic rate of glucose	Castrillon et al, 2023 (https://doi.org/10.1126/sciadv.adi7632)	SERT (map 2)	Serotonin transporter	Savli et al, 2012 (https://doi.org/10.1016/j.neuroimage.2012.07.001)
COX1	Cyclooxygenase‐1	Kim et al, 2020 (https://doi.org/10.1007/s00259‐020‐04855‐2)	SERT (map 3)	Serotonin transporter	Beliveau et al, 2017 (https://doi.org/10.1523/JNEUROSCI.2830‐16.2016)
D1	Dopamine D1 receptor	Kaller et al, 2017 (https://doi.org/10.1007/s00259‐017‐3645‐0)	SV2A	Synaptic vesicle glycoprotein 2A	Finnema et al, 2017 (https://doi.org/10.1177/0271678X17724947)
D2 (map 2)	Dopamine D2 receptor	Alakurtti et al, 2025 (https://doi.org/10.1038/jcbfm.2015.53)	VAChT	Vesicular acetylcholine transporter	Hansen et al, 2022 (https://doi.org/10.1038/s41593‐022‐01186‐3)
D2 (map 2)	Dopamine D2 receptor	Jaworska et al, 2020 (https://doi.org/10.1038/s41386‐020‐0662‐7)	VAChT (map 2)	Vesicular acetylcholine transporter	Aghourian et al, 2017 (https://doi.org/10.1016/10.1038/mp.2017.183)
DAT	Dopamine transporter	Dukart et al 2018 (https://doi.org/10.1038/s41598‐018‐22444‐0)	VAChT (map 3)	Vesicular acetylcholine transporter	Bedard et al, 2019 (https://doi.org/10.1016/j.sleep.2018.12.020)
FDOPA	Fluorodopa	Garcia‐Gomez et al, 2018 (https://doi.org/10.33588/imagendiagnostica.901.2)	VMAT2	Vesicular monoamine transporter 2	Larsen et al, 2020 (https://doi.org/10.1038/s41467‐020‐14693‐3)
GABAa5	GABA‐A receptor α5‐ component)	Lukow et al, 2022 (https://doi.org/10.1038/s42003‐022‐03268‐1)	mGluR5	Metabotropic glutamate receptor 5	Hansen et al, 2022 (https://doi.org/10.1038/s41593‐022‐01186‐3)
GABAa	GABA‐A receptor	Dukart et al, 2018 (https://doi.org/10.1038/s41598‐018‐22444‐0)	mGluR5 (map 2)	Metabotropic glutamate receptor 5	Dubois et al, 2015 (https://doi.org/10.1007/s00259‐015‐3167‐6)
GABAa (map 2)	GABA‐A receptor	Norgaard et al, 2021 (https://doi.org/10.1016/j.neuroimage.2021.117878)	mGluR5 (map 3)	Metabotropic glutamate receptor 5	Smart et al, 2019 (https://doi.org/10.1007/s00259‐018‐4252‐4)

Receptor abbreviations, full names, and references of all 44 PET‐derived neurotransmitter maps used for spatial correlations.

GM = gray matter; PET = positron emission tomography.

To illustrate the concordance of patterns of atrophy and neurotransmitter distributions, we generated threshold‐free cluster enhancement (TFCE)‐corrected *p*‐maps comparing 113 C9NEG and 113 HC at baseline with the relevant covariates. These maps were based on GM concentration data obtained from the CAT12 toolbox in MNI152 space. The analysis was performed using FSL's^15^ randomize tool with 5,000 permutations, testing for lower GM concentration in the C9NEG group using a 1‐sided test. We controlled for the following confounders; age, sex, handedness, years of education, symptom duration, and TIV. The TFCE‐corrected *p*‐maps were then overlaid on selected neurotransmitter maps with the highest spatial correlation (lowest *p*‐values). To enhance visibility, GM atrophy maps were displayed with a *p*_TFCE_ <0.10, whereas PET maps' display ranges were adjusted to maximize overlap. PET maps within the JuSpace toolbox are standardized, ranging between (0–100), but they are derived from different inputs based on original studies. These original research studies are summarized and referenced in Table 2.

## Results

A total of 145 genetically stratified patients with ALS (130 C9NEG, 15 C9POS) and 113 HCs were included (Table 1). No significant differences were observed in age or handedness between ALS and HCs, but differences existed in sex distribution and education (Table 1). Baseline ALSFRS‐r scores were lower in C9POS than in C9NEG (*t*(14.8) = 2.526, *p* = 0.023). ALSFRS‐r scores in C9NEG showed no significant longitudinal change across sessions (paired *t* tests: *p* > 0.05, Friedman test: *p* = 0.764) (Table 1).

Cross‐sectional spatial correlation analyses revealed distinct patterns of neurotransmitter associations (Fig 1, Table 3 and Table S1). In C9NEG patients, significant topological correlations (p_FDR_BH <0.05) were observed between GM volume loss and physiological 5HT1a, GABAa5, KOR expression patterns. The spatial correlations between atrophy patterns in C9NEG and GABAa5, 5HT1a, and KOR expression maps are further illustrated in Figure 2A–C. In the C9POS group, a broader spectrum of anatomical associations emerged in addition to 5HT1a, GABAa5, and KOR, including mGluR5, 5HT2a, CB1, Microglia, and MOR maps. The direct C9POS versus C9NEG GM profile contrast revealed *C9orf72*‐specific associations with Microglia, 5HT2a, mGluR5, and COX1, suggesting that neurodegeneration in GGGGC hexanucleotide expansion carriers is particularly associated with these neurotransmitter receptor distributions (Table 3 and Table S1).

**FIGURE 1:**
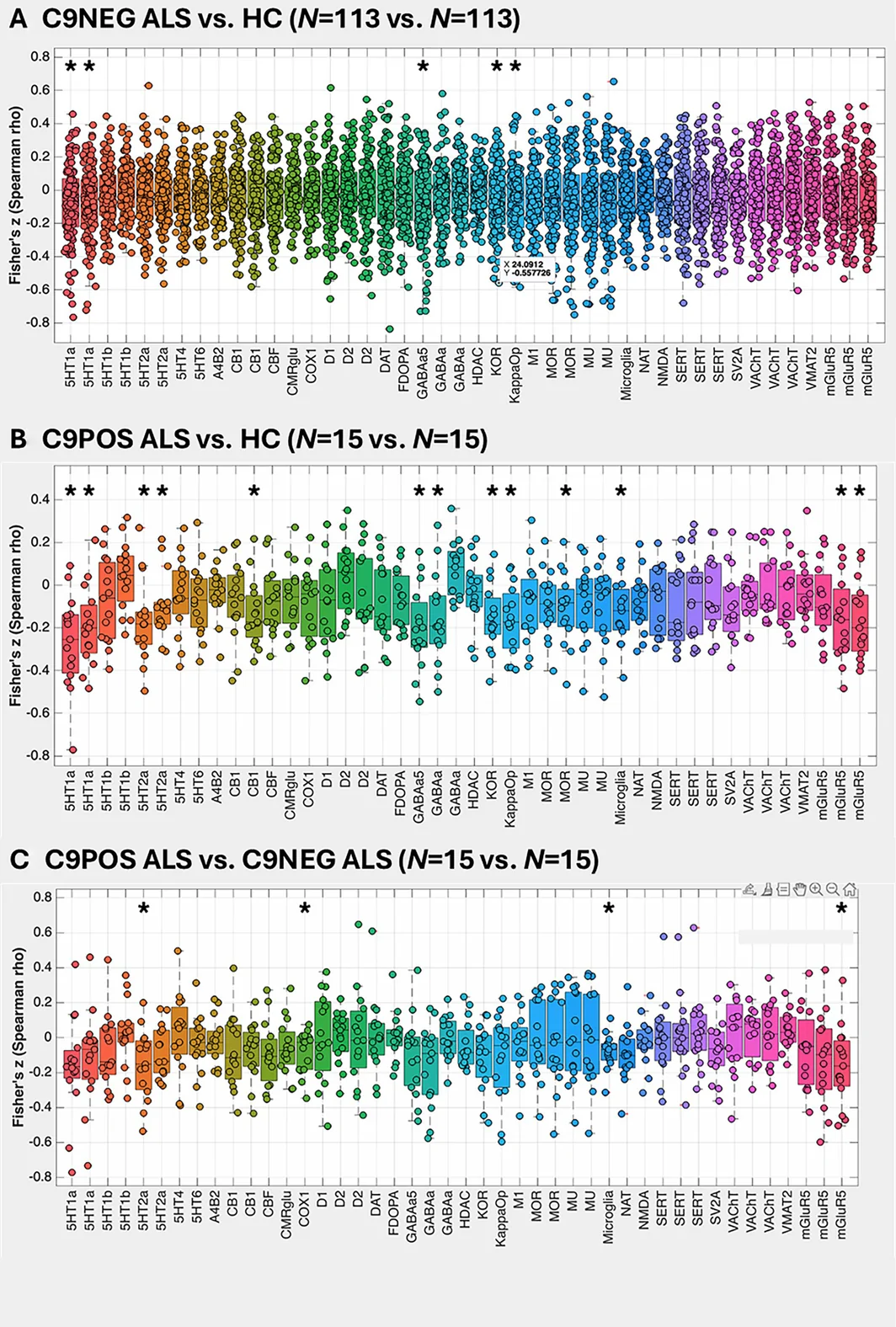
Anatomical correlations between gray matter residual volumes at baseline and neurotransmitter PET expression maps in ALS subgroups. Box plots depict the distribution of voxel‐wise Fisher's z values across all voxels for each neurotransmitter PET map in the 3 contrasts: (A) C9NEG versus HC (n = 113 vs 113), (B) C9POS versus HC (n = 15 vs 15), and (C) C9POS versus C9NEG (n = 15 vs 15). Each box represents 1 PET map, with the central line indicating the median Fisher's z, box edges the interquartile range, whiskers the range excluding outliers, and dots individual voxel values. Significant associations (p_FDR_BH <0.05) are marked with asterisks (*). ALS = amyotrophic lateral sclerosis; C9NEG = sporadic patients with ALS who tested negative for a comprehensive panel of ALS‐associated genetic variants as well as *C9orf72* GGGGCC hexanucleotide repeat expansions; C9POS = patients with ALS carrying the GGGGCC hexanucleotide repeat expansion in *C9orf72*; HC = healthy controls; Fisher's z = mean Fisher‐transformed correlation coefficient derived from voxel‐wise Spearman rho values; PET = positron emission tomography; p_FDR_BH = Benjamini‐Hochberg false discovery rate‐corrected p‐value. [Color figure can be viewed at www.annalsofneurology.org]

**TABLE 3:** Anatomical Correlations between Gray Matter Atrophy Patterns and Neurotransmitter PET Maps at Baseline

Rank	PET map	Mean Fisher's z	Median Spearman ρ	p_FDR_BH
C9NEG vs HC (baseline, n = 113 vs n = 113)				
1	5HT1a	−0.0832	−0.0645	0.0066
2	5HT1a (map 2)	−0.076	−0.0548	0.0066
3	GABAa5	−0.0776	−0.0589	0.0079
4	KOR	−0.0618	−0.0581	0.0079
5	KappaOp	−0.0674	−0.0534	0.0079
C9POS vs HC (baseline, n = 15 vs n = 15)				
1	5HT1a	−0.2651	−0.2488	0.0005
2	GABAa5	−0.1997	−0.206	0.0005
3	5HT1a (map 2)	−0.1958	−0.198	0.0005
4	mGluR5 (map 3)	−0.1639	−0.1969	0.0005
5	KOR	−0.1532	−0.182	0.0005
6	5HT2a	−0.1677	−0.1799	0.0005
7	KappaOp	−0.1647	−0.1708	0.0005
8	CB1 (2nd map)	−0.133	−0.1708	0.0005
9	Microglia	−0.1281	−0.1193	0.0005
10	mGluR5 (2nd map)	−0.1599	−0.1604	0.0026
11	GABAa	−0.1532	−0.1893	0.0068
12	MOR (map 2)	−0.1085	−0.0825	0.0278
13	5HT2a (map 2)	−0.0952	−0.1057	0.0342
C9POS vs C9NEG (baseline, n = 15 vs n = 15)				
1	Microglia	−0.0788	−0.0686	0.0044
2	5HT2a	−0.1531	−0.1502	0.011
3	mGluR5 (map 3)	−0.1603	−0.1609	0.0352
4	COX1	−0.0847	−0.0267	0.0484

Neurotransmitters ranked based on the significance of their voxel‐wise spatial correlations with atrophy patterns in specific group comparisons C9NEG vs HC, C9POS vs HC and C9POS vs C9NEG The full list of neurotransmitters are presented in Table S1. Results are first ranked by *p*‐value, then by median Spearman rho (ρ).

5HT1a = serotonin 1a receptor ALS = amyotrophic lateral sclerosis; C9POS = C9orf72‐positive ALS; C9NEG = sporadic ALS without known ALS‐associated genetic variants; CB1 = cannabinoid type 1 receptor; COX1 = cyclooxygenase‐1; GABAa = GABA‐A receptor benzodiazepine binding site; GABAa5 = GABA‐A receptor α5‐selective component; HC = healthy controls; KappaOp = kappa opioid receptor; KOR = kappa opioid receptor; mGluR5 = metabotropic glutamate receptor 5; Microglia = activated microglia; MOR = mu opioid receptor; PET = positron emission tomography; p_FDR_BH = *p*‐value adjusted for false discovery rate using the Benjamini‐Hochberg procedure.

**FIGURE 2:**
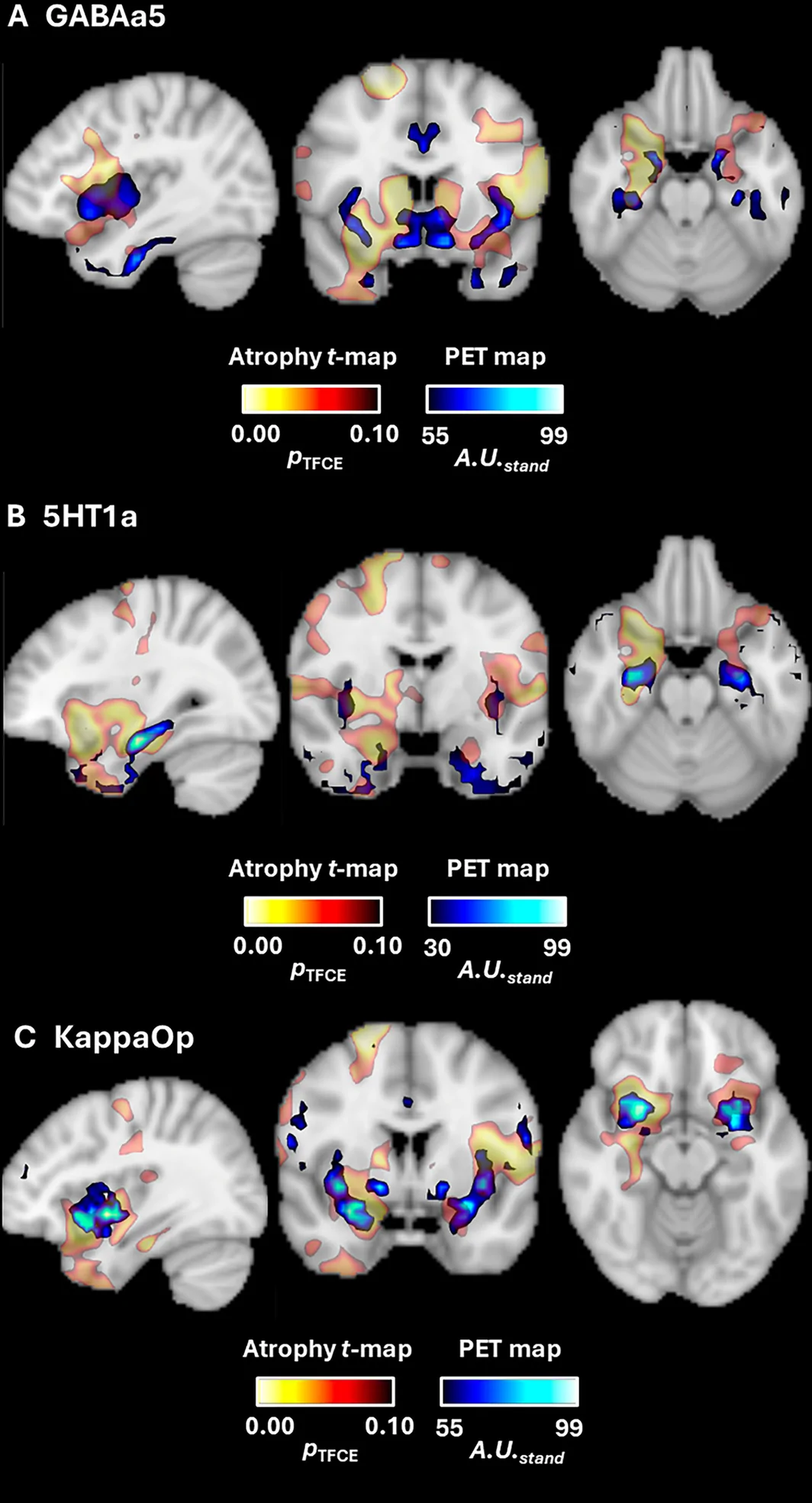
Anatomical overlap between morphometric gray matter atrophy maps and PET receptor density distributions. In A–C, TFCE‐corrected *p*‐maps depicting GM concentration reductions in C9NEG ALS compared to healthy controls is shown in hot colors (red to white) at *p*TFCE <0.10. Cool colors (blue spectrum) indicate PET‐derived neurotransmitter maps from the JuSpace toolbox, standardized between (0–100): (A) GABAa5, (B) 5HT1a, (C) KappaOp. These maps were selected because they produced the highest spatial correlations in the JuSpace baseline comparison between C9NEG and HC (see Table 3). PET map display ranges were adjusted for optimal visual overlap, with slice selection tailored to each view. 5HT1a = serotonin 1a receptor; ALS = amyotrophic lateral sclerosis; A.U. = arbitrary unit; C9NEG = sporadic patients with ALS who tested negative for a comprehensive panel of ALS‐associated genetic variants as well as C9orf72 GGGGCC hexanucleotide repeat expansions; GABAa5 = GABA‐A receptor α5‐selective component; HC = healthy controls; KappaOp = kappa opioid receptor; PET = positron emission tomography; TFCE = threshold‐free cluster enhancement. [Color figure can be viewed at www.annalsofneurology.org]

Longitudinal analyses in C9NEG revealed progressive changes in spatial correlations over time (Fig 3, Table 3, Table S3, and Fig S1). Regression of mean Fisher's z values across timepoints 1 to 4 revealed significant temporal trends (*p* < 0.005) for multiple maps, including GABAa5 (*p* = 0.0014), NAT (*p* = 0.0015), and FDOPA (*p* = 0.0021) (Table 4). Positive *r* values indicate increasing spatial associations (NAT, GABAa5), whereas negative *r* values indicate dissociation trends (FDOPA). Although these results reveal dynamic temporal associations between focal volume loss and neurotransmitter expression, it is noteworthy that p_FDR_BH values do not reach significance at later timepoints. At timepoint 1, there are 5 neurotransmitter maps exhibiting significant associations with atrophy based on p_FDR_BH <0.05, whereas on follow‐up at timepoints 2, 3, and 4, significance for spatial associations is lost for each neurotransmitter (Table S2).

**FIGURE 3:**
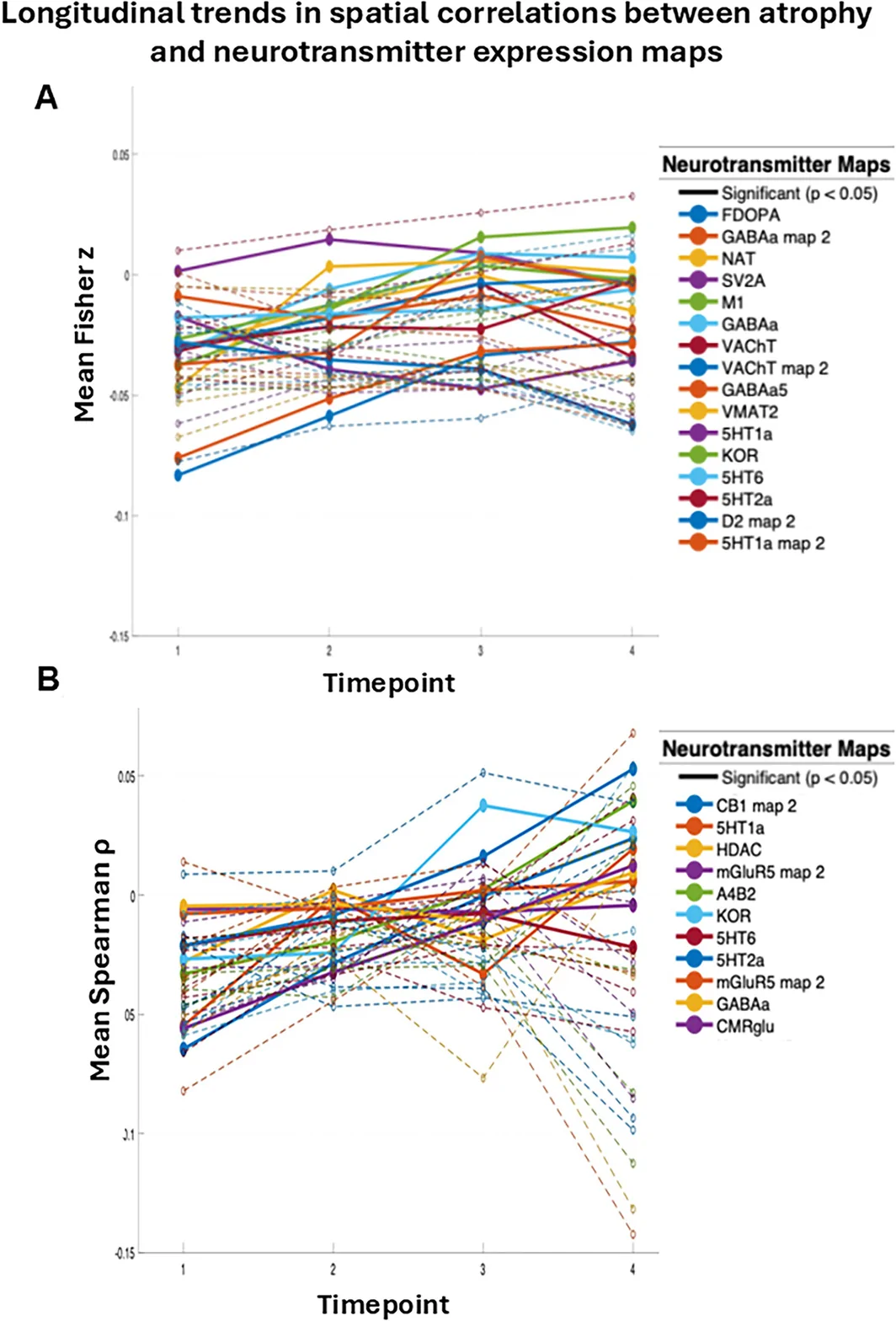
Longitudinal changes in spatial correlations between atrophy and neurotransmitter expression maps in C9NEG ALS. (A) Line plots show mean Fisher z values (y‐axis) across the 4 longitudinal timepoints for each neurotransmitter map. Significant trends (*p* < 0.05 for Pearson correlation of mean Fisher's z over time) are shown with solid lines. (B) Line plots show mean Spearman rho values (y‐axis) across the 4 longitudinal timepoints for each neurotransmitter map. Significant trends (*p* < 0.05 for Pearson correlation of mean Fisher's z over time) are shown with solid lines. ALS = amyotrophic lateral sclerosis; C9NEG = sporadic patients with ALS who tested negative for a comprehensive panel of ALS‐associated genetic variants as well as C9orf72 GGGGCC hexanucleotide repeat expansions; HC = healthy controls; Spearman rho = median voxel‐wise Spearman rank correlation coefficient; Pearson's *r* = Pearson correlation coefficient of mean Fisher's z over time; p = 2‐tailed *p*‐value of Pearson correlation (uncorrected); PET = positron emission tomography. [Color figure can be viewed at www.annalsofneurology.org]

**TABLE 4:** Longitudinal Trajectories of Anatomical Neurotransmitter Associations in the *C9orf72* Negative ALS Cohort

Rank	PET map	Pearson's *r*	*p*
1	GABAa5	0.9986	0.0014
2	NAT	0.9985	0.0015
3	FDOPA	−0.9979	0.0021

Neurotransmitter receptor populations showing progressive association (GABAa5, NAT) and dissociation (FDOPA) with patterns of atrophy over time. Statistics for all neurotransmitter maps are presented in Table S3.

ALS = amyotrophic lateral sclerosis; C9POS = C9orf72‐positive ALS; C9NEG = sporadic ALS without known ALS‐associated genetic variants; FDOPA = fluorodopa; GABAa5 = GABA‐A receptor α5‐selective component; HC = healthy controls; NAT = noradrenaline transporter; PET = positron emission tomography.

## Discussion

Our results indicate that neurodegeneration in sporadic ALS has a propensity to brain regions with high physiological GABAa5, 5HT1a, and KOR expression. In cohorts with GGGGCC hexanucleotide repeat expansions, additional predilection was detected to brain regions with high mGluR5, 5HT2a, CB1, Microglia, and MOR densities. This was confirmed by our C9POS versus C9NEG contrast demonstrating C9orf72‐specific anatomical associations with Microglia, 5HT2a, mGluR5, and COX1 concentration distributions. Longitudinal analyses revealed progressive associations with GABAa5 and norepinephrine over time. Our findings not only suggest neurotransmitter‐defined circuitry vulnerability patterns in ALS, but indicate genotype‐specific associations with specific neurotransmitter systems. The neurotransmitter‐associations revealed by this study are in line with postmortem and animal studies,^11^, ^16^, ^17^, ^18^, ^19^ but additionally, our data confirms in vivo concordance between focal neurodegeneration and receptor densities, suggesting a predilection to specific neurotransmitter‐defined networks. The relevance of linking dynamic neurodegenerative processes to specific receptors stems from the opportunity to target these specific circuits pharmacologically. It has long been suggested that because of the considerable genetic and phenotypic heterogeneity of ALS, it would be naïve to expect a single “cure” for all patients with ALS. Our genotype‐stratified analyses suggest that neurodegeneration in GGGGCC hexanucleotide carriers may be modulated by different networks.

### Disease Biology, Clinical Relevance, and Putative Pharmacological Targets

ALS has long been associated with the dysfunction of specific neurotransmitters, but the majority of these insights stem from animal models, complex neurophysiology studies,^20^ in vitro labeling studies,^16^ cerebrospinal fluid (CSF) analyses,^17^ and magnetic resonance spectroscopy (MRS).^18^ Motor system degeneration in ALS is classically linked to glutamate excitotoxicity and impaired inhibitory GABA signaling.^11^ Adenosine triphosphate and histamine secreted by microglia are thought to trigger detrimental inflammatory responses that further damage motor neurons.^19^ Reduced inhibitory glycine levels in the spinal cord are believed to make motor neurons more vulnerable to the excitotoxicity. More recent studies have consistently implicated serotonergic dysfunction.^21^ Reductions in serotonin activity is thought to catalyze disease progression by failing to inhibit glutamatergic transmission.

In our *C9orf72* negative, sporadic cohort we have identified a disease burden predilection to GABAa5, 5HT1a, and KOR mediated brain regions. GABAergic dysfunction has long been linked to ALS. Reductions in motor cortex GABA has been demonstrated in vivo by 3T 1H‐MRS^22^ and reduced GABA‐mediated inhibition has been linked to cortical hyperexcitability in mouse models using patch‐clamp recordings.^23^ α5 subunit containing GABA type A receptors are highly expressed in the hippocampus and play a crucial role in cognitive processes, synaptic plasticity, and tonic inhibition. They modulate hippocampal pyramidal neuron excitability and mediate a range of learning and memory processes.^24^ This receptor population is a key therapeutic target in autism, schizophrenia, neurodevelopmental syndromes, and Alzheimer's disease. Drug development efforts typically focus on α5‐selectivity to enhance memory without the sedating side effects of GABA‐A agonists. Positive allosteric modulators (PAMs) have been consistently investigated in ALS to enhance inhibitory function with relatively limited success.^25^ Although cerebral GABAa5 receptors are primarily evaluated in a cognitive context, they are also expressed in the spinal cord^26^ indicating a complex role in sensory‐motor control and a therapeutic target in pain modulation, spasticity management, and motor control. Serotonin 5HT1a receptor‐mediated mechanisms are also recognized in ALS with studies revealing decreased cortical binding^27^ and increased spinal cord receptor density.^21^ Both cerebrospinal fluid and serum levels of the 5‐HT precursor tryptophan are reduced in ALS.^28^ Reduced cerebral 5HT1A binding highlighted by PET studies have been primarily interpreted as neuronal loss, but these changes may also represent alterations in receptor expression or function.^21^, ^27^ It has been suggested that pathognomonic brain regions, preferentially affected in ALS, such as the motor cortex, ambiguous or hypoglossal brainstem nuclei receive ample 5‐HT innervation, whereas more resilient motoneuron groups, such as oculomotor, trochlear, and abducens nuclei, are much less sparsely innervated by 5‐HT neurons.^8^, ^21^ These observations are mirrored by our findings, demonstrating that brain regions with ample 5HT1a expression are more likely to succumb to neurodegeneration. Alterations in the serotonergic system are thought to contribute to motor neuron excitability, and 5HT1a agonists have been repeatedly proposed as putative therapeutic agents to modulate glutamate‐induced neurotoxicity.^21^ Insights from primary cortical cell cultures indicate that 5HT1A and 5HT2 receptor agonists may reduce glutamate‐induced neurotoxicity.^29^ Animal studies indicate that 5‐HT precursor 5‐hydroxytryptophan (5‐HTP) improves locomotor function and survival of transgenic SOD1 G93A mice,^30^ supporting the putative role of serotonergic pharmacological interventions in ALS. One of the common clinical manifestations of ALS is pseudobulbar affect (PBA), which has been repeatedly linked to serotonergic dysfunction and the SSRI and TCAs are commonly used first line agent to effectively ameliorate PBA‐related manifestations.^31^ Our data also suggest anatomical associations between neurodegeneration and KOR distribution. Naltrexone is a mu‐, delta‐, and kappa‐opioid receptor agonist and has been recently trialed in several neurodegenerative conditions including ALS. Low‐dose naltrexone (LDN) has been evaluated in ALS with disappointing results.^32^

In our *C9orf72* repeat expansion cohort, genotype‐specific topological associations have been identified with mGluR5, 5HT2a, CB1, Microglia, and MOR maps. Cerebral and spinal mGluR5 is upregulated in both patients with ALS and mouse models,^33^ contributing to excitotoxicity and disease progression. Therapies focusing on mGluR5 depletion have been promising in slowing disease progression in mouse model.^34^, ^35^ The activation of CB1 receptors reduces glutamate release mitigating excitotoxicity. CB1 receptors are likely to play a complex role in the pathophysiology of ALS, but reports on their expression level alterations are conflicting. Some animal model studies suggest decreased spinal expression contributing to vulnerability to excitotoxic damage,^36^ whereas others suggest increased sensitivity of cannabinoid CB1 receptors in the striatum of symptomatic mice. Delta‐9‐tetrahydrocannabinol (THC), a partial CB1 and CB2 agonist delays motor decline in SOD1 mice.^37^ Although human studies have not demonstrated a survival benefit to date,^38^ the endocannabinoid system is very likely to be involved in the pathophysiology of ALS, and CB1 receptor activation remains an active field of therapy development. Our findings support this association and suggest that focal degeneration and CB1 expression may be particularly closely linked in GGGGCC hexanucleotide repeat expansion carriers. Associations with MOR maps are of particular interest as this pathway is not typically implicated in disease biology. MOR agonists are widely used in the palliative management of ALS, but unlike serotonergic, GABAergic, anti‐glutamate, and endocannabinoid signaling systems, MU receptors are not typically associated with ALS pathophysiology. Topological associations with Microglia, however, are well in line with the neuroinflammation hypothesis of ALS. This map has been generated based on 11C‐PBR28 binding distribution, which is an 18kDa translocator protein‐targeted tracer (TSPO). TSPO is expressed at very low levels in the healthy central nervous system, but becomes dramatically upregulated by reactive microglia and astrocytes in the context of neuroinflammation.^39^ Neuroinflammation is a key aspect of ALS pathophysiology. Once regarded as a passive, downstream, post‐cell death process, it is now recognized as a key driver of neurodegeneration and promising therapeutic target.^40^ Components of the inflammatory cascade include the pro‐inflammatory, “neurotoxic” (M1) transformation of microglia, toxic cytokine producing reactive astrocytes, blood–brain barrier (BBB) dysfunction with ensuing macrophage, T cell, and natural killer (NK) infiltration, and the depletion of functional regulatory T cells. The anatomical associations between atrophy and TSPO‐derived microglial markers in our study support the notion that neuroinflammation plays an active role in neurodegeneration. *C9orf72* deficiency has been specifically associated with hyper‐reactive innate immune response and lysosomal microglia dysfunction.^41^ As microglial dysfunction is independent from primary neuronal processes,^42^ attempts to reduce neuroinflammation and restore microglial homeostasis is an active and promising field of pharmacological research.^43^ Strategies such as autophagy activation and targeting toxic species are being explored.

Our longitudinal analyses in sporadic patients revealed progressive changes over time (see Fig 3, Table 3, and Table S2) with increasing spatial associations between atrophy and NAT and GABAa5 expression and dissociation trends with FDOPA. Although these results confirm dynamic temporal changes in association strength between volume loss and neurotransmitter expression, because of the high attrition rates and ensuing drop in sample size at later timepoints, correlation strengths do not reach significance at later timepoints (Table S2). It is also noteworthy that ALS has a long presymptomatic phase during which significant pathological changes have already taken place,^1^ and the 4 timepoints of this study (12 months) only encompass a relatively short period of time in comparison to the overall duration of the disease process.

### Neuroimaging Perspectives

The reported associations with specific neurotransmitter‐mediated networks are well in line with the paradigm shift of no longer regarding ALS as the manifestation of focal degenerative changes, but its reconceptualization as a “network disorder” or “disconnection syndrome.” Recent studies have consistently demonstrated that clinical disability in ALS stems from progressive inter‐hemispheric, cortico–bulbar, cortico–basal, cortico–thalamic, and cerebro–cerebellar disconnection.^44^, ^45^, ^46^ The description of genotype‐specific vulnerability patterns and magnetic resonance imaging (MRI) signatures has gained practical relevance with the implementation of robust machine learning (ML) frameworks. ML models increasingly facilitate the interpretation of single‐subject data sets in ALS and may be helpful to categorize individual patients into relevant diagnostic, phenotypic, or prognostic categories.^47^, ^48^, ^49^

### Novelty and Limitations

Our study highlights significant anatomical association with specific receptor populations in both sporadic and *C9orf72*‐assocaited ALS. Our results not only confirm established disease mechanisms such as glutamate and GABAergic associations, they are consistent with recognized disease mechanisms such as serotonergic hypotheses, and also support emerging pathways such as endocannabinoid‐mediated neurodegeneration. Associations with Microglial markers in the *C9orf72* cohort support the role of neuroinflammatory cascades. Juxtaposition of large patient‐derived clinical data sets to physiological receptor distributions not only helps to identify potential targets for therapeutic interventions, but by ranking the strength of these associations (Table S1), it is possible to eliminate receptor populations that are less relevant to therapy development. Such analyses may help to narrow down receptor candidates for putative agonist–antagonist interventions. Our analyses reveal that certain receptor types show no spatial associations at all with neurodegenerative change and may, therefore, be less pertinent to the pathophysiology of ALS. In the *C9orf72* negative sporadic cohort, no associations were found between atrophy and the following receptor populations; CMRglu, CB1, FDOPA, VAChT, GABAa, A4B2, D2, HDAC, VMAT2, and 5HT1b. The absence of associations with D2, FDOPA, and A4B2 are of particular interest, as these systems would be relatively easily targeted with existing medications. Our results also highlight the specificity of association with receptor subtypes. Although 5HT1a expression is very closely associated with atrophy (p_FDR_BH = 0.0066), 5HT1b map2 exhibits a p_FDR_BH value of just 0.1179. Similarly, the expression of GABAa5 is closely linked to neurodegeneration (p_FDR_BH = 0.0079), whereas GABAa does not show topological associations at all (p_FDR_BH = 0.1484). CB1 seems specifically associated with *C9orf72* (p_FDR_BH = 0.0005) and not with sporadic disease (p_FDR_BH = 0.0621), which may explain the inconsistent reports of therapies targeting the endocannabinoid system.^32^ Overall, our analyses may help to identify pharmacologically‐relevant receptor populations in ALS and limit research efforts in modulating networks, which are much less relevant to the pathophysiology of ALS. From a diagnostic and clinical trial perspective, neurotransmitter‐associations may inform ligand development efforts for next generation PET tracers to detect, confirm, and monitor ALS‐specific signatures early in the course of the disease.

A key limitation of our study is that neurotransmitter and metabolic markers have not been measured in the serum and CSF of the patients. Furthermore, dedicated metabolic imaging modalities such as MRS^50^ or PET with specific ligands have not been performed. However, our MRI‐based approach has enabled the evaluation of associations with 44 neurotransmitter maps, which would not have been possible with a specific PET ligand. It is noteworthy, that the vast majority of published ALS PET studies are fluorodeoxyglucose‐PET studies, using a tracer that is a glucose analogue, internalized by glucose transporter 1 (GLUT1), thereby an excellent marker of focal metabolism, but not indicative of specific receptor families. A further limitation of our study is the absence of postmortem analyses to confirm atrophy patterns, the depletion of specific neuronal populations and assessment of receptor expression levels by in vitro labeling.^16^ Despite the multi–time point longitudinal design and the large initial cohort size, the high longitudinal attrition rate impacted sample sizes at the later timepoints hindering the interpretation of late‐stage correlations.

## Conclusions

Our study confirms established glutamate‐mediated and GABA‐modulated degeneration, supports serotonergic hypotheses, implicates the endocannabinoid system, and points to Microglia mediated neuroinflammation. We describe neurotransmitter expression associated degeneration in a large, sporadic, “gene negative cohort,” and present *C9orf72*‐specific associations. Confirming the role of specific neurotransmitter populations in the pathophysiology in ALS and highlighting the lack of impact of others, which may be relevant to targeted therapy development.

## Author Contributions

M.T., W.F.S., J.L., E.L.T., A.T., J.K., and P.B. contributed to the conception and design of the study; M.T., W.F.S., J.L., J.C.H., M.A.D., R.L.Mc.L., O.H., E.L.T., A.T., J.K., and P.B. contributed to the acquisition and analysis of data; M.T., W.F.S., J.L., E.L.T., J.K., and P.B. contributed to drafting the text or preparing the figures.

## Potential Conflicts of Interest

The authors declare no conflicts of interest.

## Supporting information


**Figure S1.** Horizontal bar plot of absolute Pearson's r values (correlation strength over time) for each neurotransmitter map, ranked from highest to lowest. Significant trends (*p* < 0.05) are marked with *.

**Table S1.** Anatomical correlations between grey matter atrophy patterns and neurotransmitter receptor expression maps at baseline.

**Table S2.** Longitudinal spatial correlations between grey matter atrophy patterns and all neurotransmitter receptor maps in C9NEG vs. HC.

**Table S3.** Longitudinal correlation of Mean Fisher's *z* values across timepoints in C9NEG ALS.

## Data Availability

Clinical, genetic, and neuroimaging data of individual patients cannot be made available because of institutional regulations and departmental policies, but additional information on data processing pipelines, and methods can be requested from the corresponding author.
