# Supplementary material for: Neurotransmitter‐Defined Degeneration Patterns in Sporadic and C9orf72‐Associated Amyotrophic Lateral Sclerosis: Predilection to GABAergic, Serotonergic, Opioid, Glutamatergic, Endocannabinoid, and Microglial Systems—Implications for Therapy Development
Source: Ann Neurol. 2026 Jul 21;100(5):948–61. doi: 10.1002/ana.78316 (PMC13688049; doi:10.1002/ana.78316)
Supplement: Supplementary file 1 [file ANA-100-948-s001.docx]

**Supplementary Table 1.** Anatomical correlations between grey matter atrophy patterns and neurotransmitter receptor expression maps at baseline.

| Rank | PET Map | Mean Fisher's z | Median Spearman ρ | p_FDR_BH |
| --- | --- | --- | --- | --- |
| C9NEG vs. HC (Baseline, *N*=113 vs. *N*=113) | | | | |
| 1 | 5HT1a | -0.0832 | -0.0645 | 0.0066* |
| 2 | 5HT1a (map 2) | -0.076 | -0.0548 | 0.0066* |
| 3 | GABAa5 | -0.0776 | -0.0589 | 0.0079* |
| 5 | KOR | -0.0618 | -0.0581 | 0.0079* |
| 4 | KappaOp | -0.0674 | -0.0534 | 0.0079* |
| 6 | MOR | -0.053 | -0.0661 | 0.0852 |
| 7 | mGluR5 | -0.0514 | -0.0464 | 0.0645 |
| 8 | mGluR5 | -0.0496 | -0.0821 | 0.0621 |
| 9 | MU | -0.0477 | -0.0479 | 0.1161 |
| 10 | CB1 | -0.0469 | -0.0559 | 0.0621 |
| 11 | MU | -0.0458 | -0.0413 | 0.1161 |
| 12 | M1 | -0.0454 | -0.0394 | 0.0565 |
| 13 | SERT | -0.0431 | -0.0539 | 0.1161 |
| 14 | MOR | -0.0424 | -0.0579 | 0.1161 |
| 15 | SERT | -0.042 | -0.043 | 0.1161 |
| 16 | Microglia | -0.0387 | -0.0466 | 0.0873 |
| 17 | 5HT2a | -0.0378 | -0.0331 | 0.1161 |
| 18 | D2 | -0.0372 | -0.0354 | 0.1466 |
| 19 | SV2A | -0.0368 | -0.0345 | 0.0845 |
| 20 | SERT | -0.0348 | -0.0169 | 0.1161 |
| 21 | 5HT2a | -0.0322 | -0.0268 | 0.1161 |
| 22 | 5HT4 | -0.0316 | -0.0212 | 0.1161 |
| 23 | DAT | -0.0313 | -0.0504 | 0.2593 |
| 24 | GABAa | -0.031 | -0.039 | 0.1484 |
| 25 | 5HT1b | -0.0308 | -0.028 | 0.1179 |
| 26 | 5HT6 | -0.0303 | -0.0212 | 0.1161 |
| 27 | COX1 | -0.0294 | -0.0232 | 0.1161 |
| 28 | D1 | -0.0279 | -0.0205 | 0.2643 |
| 29 | VAChT | -0.0269 | -0.0507 | 0.2643 |
| 30 | CBF | -0.0267 | -0.0183 | 0.203 |
| 31 | NMDA | -0.0243 | -0.0328 | 0.1484 |
| 32 | NAT | -0.0229 | -0.0113 | 0.1626 |
| 33 | FDOPA | -0.0211 | -0.0362 | 0.3391 |
| 34 | mGluR5 | -0.021 | -0.0179 | 0.3188 |
| 35 | CMRglu | -0.0178 | -0.0225 | 0.2928 |
| 36 | CB1 | -0.0174 | -0.0046 | 0.454 |
| 37 | VAChT | -0.0167 | -0.0459 | 0.5221 |
| 38 | VAChT | -0.0116 | -0.0305 | 0.6144 |
| 39 | A4B2 | -0.0089 | -0.0078 | 0.5935 |
| 40 | D2 | -0.005 | -0.0216 | 0.8027 |
| 41 | HDAC | -0.0048 | 0.0139 | 0.7727 |
| 42 | VMAT2 | 0.0008 | -0.0083 | 0.9711 |
| 43 | 5HT1b (map2) | 0.0015 | -0.0059 | 0.9311 |
| 44 | GABAa | 0.01 | 0.0087 | 0.6032 |
| C9POS vs. HC (Baseline, *N*=15 vs. *N*=15) | | | | |
| 1 | 5HT1a | -0.2651 | -0.2488 | 0.0005 * |
| 2 | GABAa5 | -0.1997 | -0.206 | 0.0005 * |
| 3 | 5HT1a (map 2) | -0.1958 | -0.198 | 0.0005 * |
| 4 | mGluR5 (map 3) | -0.1639 | -0.1969 | 0.0005 * |
| 5 | KOR | -0.1532 | -0.182 | 0.0005 * |
| 6 | 5HT2a | -0.1677 | -0.1799 | 0.0005 * |
| 7 | KappaOp | -0.1647 | -0.1708 | 0.0005 * |
| 8 | CB1 (map 2) | -0.133 | -0.1708 | 0.0005 * |
| 9 | Microglia | -0.1281 | -0.1193 | 0.0005 * |
| 10 | mGluR5 (map 2) | -0.1599 | -0.1604 | 0.0026 * |
| 11 | GABAa | -0.1532 | -0.1893 | 0.0068 * |
| 12 | MOR (map 2) | -0.1085 | -0.0825 | 0.0278 * |
| 13 | 5HT2a (map 2) | -0.0952 | -0.1057 | 0.0342 * |
| 14 | SV2A | -0.0978 | -0.1386 | 0.0517 |
| 15 | MOR | -0.09 | -0.0888 | 0.0517 |
| 16 | NAT | -0.0808 | -0.078 | 0.0517 |
| 17 | FDOPA | -0.072 | -0.0859 | 0.0956 |
| 18 | COX1 | -0.0899 | -0.0408 | 0.1152 |
| 19 | SERT | -0.0967 | -0.1722 | 0.1192 |
| 20 | CBF | -0.0747 | -0.0745 | 0.1275 |
| 21 | DAT | -0.0788 | -0.1463 | 0.171 |
| 22 | mGluR5 | -0.0734 | -0.1034 | 0.171 |
| 23 | M1 | -0.0808 | -0.054 | 0.171 |
| 24 | NMDA | -0.0667 | -0.0283 | 0.171 |
| 25 | 5HT6 | -0.0691 | -0.07 | 0.1795 |
| 26 | D1 | -0.0749 | -0.0694 | 0.2249 |
| 27 | 5HT1b | -0.0715 | -0.0603 | 0.2249 |
| 28 | CMRglu | -0.0589 | -0.0547 | 0.2249 |
| 29 | D2 (map 3) | -0.0714 | -0.1278 | 0.3266 |
| 30 | D2 (map 2) | -0.0714 | -0.1277 | 0.3266 |
| 31 | SERT (map 2) | -0.0589 | -0.0792 | 0.3674 |
| 32 | 5HT4 | -0.0462 | -0.0617 | 0.373 |
| 33 | VAChT | -0.0354 | -0.0436 | 0.373 |
| 34 | VAChT | -0.0354 | -0.0436 | 0.373 |
| 35 | A4B2 | -0.0322 | -0.0217 | 0.373 |
| 36 | VAChT (map 3) | -0.0394 | -0.0064 | 0.373 |
| 37 | VAChT (map 3) | -0.0394 | -0.0064 | 0.373 |
| 38 | 5HT1b (map 2) | 0.0741 | 0.0456 | 0.373 |
| 39 | SERT (map 3) | -0.0423 | -0.0961 | 0.3934 |
| 40 | HDAC | -0.0334 | -0.0301 | 0.4401 |
| 41 | D2 | 0.0328 | 0.0605 | 0.4939 |
| 42 | VAChT (map 2) | -0.0175 | -0.0885 | 0.6829 |
| 43 | VAChT (map 2) | -0.0175 | -0.0885 | 0.6829 |
| 44 | VMAT2 | -0.0155 | -0.0413 | 0.7094 |
| C9POS vs. C9NEG (Baseline, *N*=15 vs. *N*=15) | | | | |
| 1 | Microglia | -0.0788 | -0.0686 | 0.0044 * |
| 2 | 5HT2a | -0.1531 | -0.1502 | 0.011 * |
| 3 | mGluR5 (map) 3 | -0.1603 | -0.1609 | 0.0352 * |
| 4 | COX1 | -0.0847 | -0.0267 | 0.0484 * |
| 5 | GABAa | -0.1566 | -0.0902 | 0.0651 |
| 6 | NAT | -0.094 | -0.1021 | 0.1078 |
| 7 | 5HT1a | -0.1644 | -0.1651 | 0.1111 |
| 8 | KOR | -0.1112 | -0.0825 | 0.1111 |
| 9 | CBF | -0.0943 | -0.1078 | 0.1914 |
| 10 | SV2A | -0.0798 | -0.064 | 0.1924 |
| 11 | CB1 (map 2) | -0.0788 | -0.0444 | 0.228 |
| 12 | mGluR5 | -0.125 | -0.0974 | 0.2342 |
| 13 | GABAa5 | -0.1033 | -0.046 | 0.2388 |
| 14 | KappaOp | -0.111 | -0.045 | 0.249 |
| 15 | 5HT1a (map 2) | -0.1122 | -0.0677 | 0.3311 |
| 16 | VMAT2 | 0.0421 | 0.0307 | 0.3351 |
| 17 | CB1 | -0.081 | -0.114 | 0.3674 |
| 18 | CMRglu | -0.0578 | -0.0729 | 0.4057 |
| 19 | 5HT1b | -0.0744 | -0.1378 | 0.4109 |
| 20 | HDAC | -0.0445 | -0.086 | 0.4525 |
| 21 | mGluR5 (map 2) | -0.0707 | -0.0507 | 0.4666 |
| 22 | M1 | -0.0478 | -0.0211 | 0.6326 |
| 23 | 5HT6 | -0.042 | -0.0283 | 0.6816 |
| 24 | NMDA | -0.0267 | -0.0389 | 0.8527 |
| 25 | VAChT (map 3) | 0.0311 | 0.0202 | 0.8527 |
| 26 | SERT (map 3) | 0.0351 | 0.0229 | 0.9272 |
| 27 | VAChT (map 2) | 0.0214 | 0.0769 | 0.9272 |
| 28 | A4B2 | -0.0178 | -0.0288 | 0.9412 |
| 29 | MOR (map 2) | -0.0289 | -0.0289 | 0.9537 |
| 30 | 5HT1b (map 2) | 0.0222 | 0.0278 | 0.9537 |
| 31 | 5HT4 | 0.0251 | 0.0543 | 0.9537 |
| 32 | MOR | -0.0111 | -0.0435 | 0.9948 |
| 33 | MOR (map 2) | -0.011 | -0.0435 | 0.9948 |
| 34 | D1 | -0.0005 | -0.0283 | 0.9948 |
| 35 | MU | -0.007 | -0.026 | 0.9948 |
| 36 | GABAa5 (map 2) | -0.0012 | -0.0209 | 0.9948 |
| 37 | D2 (map 2) | 0.0016 | -0.0151 | 0.9948 |
| 38 | MU (map 2) | -0.0082 | -0.0133 | 0.9948 |
| 39 | DAT | 0.0138 | -0.0027 | 0.9948 |
| 40 | SERT (map 2) | 0.012 | -0.0016 | 0.9948 |
| 41 | SERT | -0.003 | 0 | 0.9948 |
| 42 | FDOPA | 0.0078 | 0.0245 | 0.9948 |
| 43 | D2 | 0.0043 | 0.0434 | 0.9948 |
| 44 | VAChT | 0.0184 | 0.0612 | 0.9948 |

Neurotransmitters ranked based on the significance of their voxel-wise spatial correlations with atrophy patterns in specific group comparisons C9NEG vs. HC, C9POS vs. HC and C9POS vs. C9NEG Significant comparisons (after FDR-corrections, *p* < .05) are highlighted with *. Results are ranked by p-value first, then by Median Spearman ρ. For more details and abbreviations of all neurotransmitter maps, please see the main manuscript.

Abbreviations: ALS: amyotrophic lateral sclerosis, C9POS: C9orf72-positive ALS, C9NEG: sporadic ALS without known ALS-associated genetic variants, HC: healthy controls, PET: positron emission tomography, p_FDR_BH: p-value adjusted for false discovery rate using the Benjamini-Hochberg procedure
