# Supplementary material for: Neurotransmitter‐Defined Degeneration Patterns in Sporadic and C9orf72‐Associated Amyotrophic Lateral Sclerosis: Predilection to GABAergic, Serotonergic, Opioid, Glutamatergic, Endocannabinoid, and Microglial Systems—Implications for Therapy Development
Source: Ann Neurol. 2026 Jul 21;100(5):948–61. doi: 10.1002/ana.78316 (PMC13688049; doi:10.1002/ana.78316)
Supplement: Supplementary file 2 [file ANA-100-948-s002.docx]

**Supplementary Table 3.** Longitudinal correlation of Mean Fisher’s z values across time points in C9NEG ALS

| Rank | PET Map | Pearson’s r | p-value |
| --- | --- | --- | --- |
| 1 | GABAa5 (map 2) | 0.9986 | 0.0014 * |
| 2 | NAT | 0.9985 | 0.0015 * |
| 3 | FDOPA | -0.9979 | 0.0021 * |
| 4 | SV2A | 0.9948 | 0.0052 |
| 5 | mGluR5 | 0.9902 | 0.0098 |
| 6 | M1 | 0.9891 | 0.0109 |
| 7 | GABAa5 | 0.9845 | 0.0155 |
| 8 | VAChT | -0.979 | 0.021 |
| 9 | mGluR5 (map 3) | 0.9777 | 0.0223 |
| 10 | VAChT (map 2) | -0.9766 | 0.0234 |
| 11 | 5HT1a | 0.9733 | 0.0267 |
| 12 | GABAa | 0.9728 | 0.0272 |
| 13 | KOR | 0.9718 | 0.0282 |
| 14 | VMAT2 | -0.9697 | 0.0303 |
| 15 | 5HT6 | 0.9678 | 0.0322 |
| 16 | 5HT2a | 0.966 | 0.034 |
| 17 | 5HT1a (map 2) | 0.9573 | 0.0427 |
| 18 | VAChT (map 3) | -0.9466 | 0.0534 |
| 19 | D1 | -0.9265 | 0.0735 |
| 20 | SERT | -0.9195 | 0.0805 |
| 21 | 5HT2a (map 2) | 0.9028 | 0.0972 |
| 22 | CMRglu | 0.8939 | 0.1061 |
| 23 | CBF | 0.8742 | 0.1258 |
| 24 | COX1 | 0.8588 | 0.1412 |
| 25 | KappaOp | 0.8489 | 0.1511 |
| 26 | DAT | -0.837 | 0.163 |
| 27 | mGluR5 (map 2) | 0.8299 | 0.1701 |
| 28 | MOR | -0.7743 | 0.2257 |
| 29 | D2 (map 2) | 0.7232 | 0.2768 |
| 30 | CB1 | 0.712 | 0.288 |
| 31 | MU | -0.6397 | 0.3603 |
| 32 | 5HT1b | 0.6266 | 0.3734 |
| 33 | SERT (map 2) | -0.6155 | 0.3845 |
| 34 | A4B2 | -0.6136 | 0.3864 |
| 35 | SERT (map 3) | -0.6026 | 0.3974 |
| 36 | CB1 (map 2) | 0.4778 | 0.5222 |
| 37 | NMDA | -0.3842 | 0.6158 |
| 38 | MU | -0.2659 | 0.7341 |
| 39 | 5HT1b (map 2) | -0.2214 | 0.7786 |
| 40 | MOR (map 2) | -0.218 | 0.782 |
| 41 | D2 | 0.1848 | 0.8152 |
| 42 | 5HT4 | 0.0684 | 0.9316 |
| 43 | HDAC | -0.0564 | 0.9436 |
| 44 | Microglia | -0.0528 | 0.9472 |

This supplementary table presents the results of the pseudo-longitudinal correlation analysis between mean Fisher’s z values (from the JuSpace spatial correlation of gray matter residuals with each neurotransmitter PET map) across four time points (inter-scan-interval: 4 months) in the C9NEG subgroup compared to baseline healthy controls (HC). Results are ranked by p-value (ascending) first, then by Pearson’s *r* (descending). Significant p-values (*p* < .05) are highlighted with *. For more details and abbreviations of all neurotransmitter maps, see the main manuscript.

Abbreviations: ALS: amyotrophic lateral sclerosis, C9POS: C9orf72-positive ALS, C9NEG: sporadic ALS without known ALS-associated genetic variants, HC: healthy controls, PET: positron emission tomography
