# Supplementary material for: Neurotransmitter‐Defined Degeneration Patterns in Sporadic and C9orf72‐Associated Amyotrophic Lateral Sclerosis: Predilection to GABAergic, Serotonergic, Opioid, Glutamatergic, Endocannabinoid, and Microglial Systems—Implications for Therapy Development
Source: Ann Neurol. 2026 Jul 21;100(5):948–61. doi: 10.1002/ana.78316 (PMC13688049; doi:10.1002/ana.78316)
Supplement: Supplementary file 3 [file ANA-100-948-s003.docx]

**Supplementary Figure 1**. Horizontal bar plot of absolute Pearson’s r values (correlation strength over time) for each neurotransmitter map, ranked from highest to lowest. Significant trends (p < 0.05) are marked with *.
