# Supplementary material for: Neurotransmitter‐Defined Degeneration Patterns in Sporadic and C9orf72‐Associated Amyotrophic Lateral Sclerosis: Predilection to GABAergic, Serotonergic, Opioid, Glutamatergic, Endocannabinoid, and Microglial Systems—Implications for Therapy Development
Source: Ann Neurol. 2026 Jul 21;100(5):948–61. doi: 10.1002/ana.78316 (PMC13688049; doi:10.1002/ana.78316)
Supplement: Supplementary file 4 [file ANA-100-948-s004.docx]

**Supplementary Table 2.** Longitudinal spatial correlations between grey matter atrophy patterns and all neurotransmitter receptor maps in C9NEG vs. HC.

| Rank | PET Map | Mean Fisher's z | Median Spearman ρ | p_FDR_BH |
| --- | --- | --- | --- | --- |
| Baseline: C9NEG vs. HC (*N*=113 vs. *N*=113) | | | | |
| 1 | 5HT1a | -0.0832 | -0.0645 | 0.0066 |
| 2 | 5HT1a (map 2) | -0.076 | -0.0548 | 0.0066 |
| 3 | GABAa5 | -0.0776 | -0.0589 | 0.0079 |
| 4 | KOR | -0.0618 | -0.0581 | 0.0079 |
| 5 | KappaOp | -0.0674 | -0.0534 | 0.0079 |
| 6 | MOR (map 2) | -0.053 | -0.0661 | 0.0852 |
| 7 | mGluR5 (map 2) | -0.0514 | -0.0464 | 0.0645 |
| 8 | mGluR5 (map 3) | -0.0496 | -0.0821 | 0.0621 |
| 9 | MU | -0.0477 | -0.0479 | 0.1161 |
| 10 | CB1 | -0.0469 | -0.0559 | 0.0621 |
| 11 | MU (map 2) | -0.0458 | -0.0413 | 0.1161 |
| 12 | M1 | -0.0454 | -0.0394 | 0.0565 |
| 13 | SERT | -0.0431 | -0.0539 | 0.1161 |
| 14 | MOR | -0.0424 | -0.0579 | 0.1161 |
| 15 | SERT (map 2) | -0.042 | -0.043 | 0.1161 |
| 16 | Microglia | -0.0387 | -0.0466 | 0.0873 |
| 17 | 5HT2a | -0.0378 | -0.0331 | 0.1161 |
| 18 | D2 (map 2) | -0.0372 | -0.0354 | 0.1466 |
| 19 | SV2A | -0.0368 | -0.0345 | 0.0845 |
| 20 | SERT (map 3) | -0.0348 | -0.0169 | 0.1161 |
| 21 | 5HT2a (map 2) | -0.0322 | -0.0268 | 0.1161 |
| 22 | 5HT4 | -0.0316 | -0.0212 | 0.1161 |
| 23 | DAT | -0.0313 | -0.0504 | 0.2593 |
| 24 | GABAa5 | -0.031 | -0.039 | 0.1484 |
| 25 | 5HT1b | -0.0308 | -0.028 | 0.1179 |
| 26 | 5HT6 | -0.0303 | -0.0212 | 0.1161 |
| 27 | COX1 | -0.0294 | -0.0232 | 0.1161 |
| 28 | D1 | -0.0279 | -0.0205 | 0.2643 |
| 29 | VAChT | -0.0269 | -0.0507 | 0.2643 |
| 30 | CBF | -0.0267 | -0.0183 | 0.203 |
| 31 | NMDA | -0.0243 | -0.0328 | 0.1484 |
| 32 | NAT | -0.0229 | -0.0113 | 0.1626 |
| 33 | FDOPA | -0.0211 | -0.0362 | 0.3391 |
| 34 | mGluR5 | -0.021 | -0.0179 | 0.3188 |
| 35 | CMRglu | -0.0178 | -0.0225 | 0.2928 |
| 36 | CB1 (map 2) | -0.0174 | -0.0046 | 0.454 |
| 37 | VAChT (map 3) | -0.0167 | -0.0459 | 0.5221 |
| 38 | VAChT (map 2) | -0.0116 | -0.0305 | 0.6144 |
| 39 | A4B2 | -0.0089 | -0.0078 | 0.5935 |
| 40 | D2 | -0.005 | -0.0216 | 0.8027 |
| 41 | HDAC | -0.0048 | 0.0139 | 0.7727 |
| 42 | VMAT2 | 0.0008 | -0.0083 | 0.9711 |
| 43 | 5HT1b (map 2) | 0.0015 | -0.0059 | 0.9311 |
| 44 | GABAa | 0.01 | 0.0087 | 0.6032 |
| Timepoint 2: C9NEG vs. HC (*N*=72 vs. *N*=72) | | | | |
| 1 | GABAa | -0.0629 | -0.0402 | 0.351 |
| 2 | KappaOp | -0.0468 | -0.0337 | 0.351 |
| 3 | SERT (map 2) | -0.0427 | -0.0335 | 0.351 |
| 4 | CB1 (map 2) | -0.0392 | -0.0323 | 0.351 |
| 5 | 5HT1a | -0.0586 | -0.0286 | 0.351 |
| 6 | SERT | -0.0466 | -0.0281 | 0.351 |
| 7 | Microglia | -0.0416 | -0.0261 | 0.351 |
| 8 | MOR (map 2) | -0.0491 | -0.0246 | 0.351 |
| 9 | VAChT | -0.0411 | -0.0228 | 0.351 |
| 10 | KOR | -0.0433 | -0.0169 | 0.351 |
| 11 | MU | -0.047 | -0.013 | 0.351 |
| 12 | MOR | -0.0438 | -0.0061 | 0.351 |
| 13 | 5HT1a (map 2) | -0.0514 | -0.0009 | 0.351 |
| 14 | DAT | -0.0468 | -0.0352 | 0.3567 |
| 15 | MU (map 2) | -0.044 | 0.0002 | 0.357 |
| 16 | VAChT (map 3) | -0.0405 | -0.0308 | 0.3616 |
| 17 | D1 | -0.0353 | -0.0468 | 0.3742 |
| 18 | mGluR5 (map 3) | -0.032 | -0.0443 | 0.3742 |
| 19 | M1 | -0.0296 | -0.0429 | 0.3742 |
| 20 | VAChT (map 2) | -0.0332 | -0.0299 | 0.3742 |
| 21 | mGluR5 (map 2) | -0.0341 | -0.0241 | 0.3742 |
| 22 | SERT (map 3) | -0.031 | -0.0385 | 0.3874 |
| 23 | NMDA | -0.0216 | -0.0307 | 0.3958 |
| 24 | SV2A | -0.0232 | -0.0115 | 0.3958 |
| 25 | FDOPA | -0.0284 | -0.01 | 0.3958 |
| 26 | A4B2 | -0.0183 | -0.0054 | 0.3958 |
| 27 | COX1 | -0.0217 | -0.0219 | 0.4084 |
| 28 | D2 (map 2) | -0.0323 | -0.0171 | 0.4084 |
| 29 | 5HT6 | -0.0181 | -0.0086 | 0.489 |
| 30 | CMRglu | -0.0161 | -0.0127 | 0.5237 |
| 31 | VMAT2 | -0.0216 | -0.0007 | 0.5237 |
| 32 | GABAa5 (map 2) | 0.0186 | 0.0101 | 0.5273 |
| 33 | 5HT1b (map 2) | 0.0146 | -0.0057 | 0.5542 |
| 34 | 5HT4 | -0.0177 | -0.0109 | 0.5898 |
| 35 | GABAa5 | -0.0176 | -0.0061 | 0.5898 |
| 36 | 5HT2a | -0.0145 | -0.0196 | 0.6376 |
| 37 | CBF | -0.0126 | -0.0127 | 0.6376 |
| 38 | HDAC | -0.0093 | -0.0067 | 0.6376 |
| 39 | 5HT1b | -0.0129 | 0.002 | 0.6376 |
| 40 | mGluR5 | -0.0115 | -0.0153 | 0.6616 |
| 41 | NAT | -0.0078 | -0.0026 | 0.7099 |
| 42 | D2 (map 2) | -0.0062 | 0.0033 | 0.7883 |
| 43 | 5HT2a (map 2) | -0.0059 | -0.0242 | 0.7937 |
| 44 | CB1 | 0.0034 | -0.0036 | 0.8791 |
| Timepoint 3: C9NEG vs. HC (*N*=47 vs. *N*=47) | | | | |
| 1 | Microglia | -0.0476 | -0.0767 | 0.5858 |
| 2 | MOR (map 2) | -0.0475 | -0.0472 | 0.5858 |
| 3 | MOR | -0.0435 | -0.042 | 0.5858 |
| 4 | MU (map 2) | -0.0438 | -0.0383 | 0.5858 |
| 5 | GABAa | -0.0596 | -0.0366 | 0.5858 |
| 6 | VAChT (map 3) | -0.0403 | -0.0296 | 0.5858 |
| 7 | SERT | -0.0475 | -0.0285 | 0.5858 |
| 8 | MU | -0.0431 | -0.0223 | 0.5858 |
| 9 | FDOPA | -0.0372 | -0.0219 | 0.5858 |
| 10 | SERT (map 2) | -0.0365 | -0.021 | 0.5858 |
| 11 | VAChT | -0.0461 | -0.0194 | 0.5858 |
| 12 | CB1 (map 2) | -0.0474 | -0.0109 | 0.5858 |
| 13 | VAChT (map 2) | -0.0389 | -0.0036 | 0.5858 |
| 14 | KOR | -0.0401 | 0.0016 | 0.5858 |
| 15 | D1 | -0.0391 | -0.0432 | 0.586 |
| 16 | KappaOp | -0.0394 | 0.0057 | 0.6328 |
| 17 | DAT | -0.0391 | 0.0137 | 0.6328 |
| 18 | SERT (map 3) | -0.0274 | -0.0392 | 0.7102 |
| 19 | GABAa5 (map 2) | 0.0257 | 0.0513 | 0.7102 |
| 20 | COX1 | -0.0226 | -0.0159 | 0.7297 |
| 21 | VMAT2 | -0.0257 | -0.0269 | 0.8005 |
| 22 | 5HT1a (map 2) | -0.0318 | -0.0331 | 0.8309 |
| 23 | 5HT1a | -0.0335 | -0.0005 | 0.8309 |
| 24 | M1 | -0.0187 | -0.0038 | 0.8402 |
| 25 | CMRglu | -0.0145 | 0.0004 | 0.8402 |
| 26 | SV2A | -0.0155 | 0.0012 | 0.8402 |
| 27 | NMDA | -0.0124 | -0.0141 | 0.8602 |
| 28 | 5HT2a | 0.0156 | 0.0024 | 0.8783 |
| 29 | HDAC | -0.0109 | -0.0205 | 0.9092 |
| 30 | D2 (map 3) | -0.0148 | -0.0101 | 0.9404 |
| 31 | 5HT1b (map 2) | 0.009 | -0.0072 | 0.9404 |
| 32 | mGluR5 (map 3) | -0.0091 | -0.0072 | 0.9404 |
| 33 | A4B2 | -0.0085 | 0.002 | 0.9404 |
| 34 | D2 (map 2) | 0.0076 | 0.0131 | 0.9404 |
| 35 | 5HT2a (map 2) | 0.0088 | 0.0376 | 0.9404 |
| 36 | CBF | 0.0037 | -0.0293 | 0.9429 |
| 37 | mGluR5 (map 2) | -0.0054 | -0.0124 | 0.9429 |
| 38 | CB1 | 0.0056 | -0.011 | 0.9429 |
| 39 | 5HT4 | -0.0036 | -0.0077 | 0.9429 |
| 40 | mGluR5 | 0.0037 | -0.0029 | 0.9429 |
| 41 | GABAa5 | 0.0074 | -0.0006 | 0.9429 |
| 42 | 5HT6 | -0.0038 | 0.0162 | 0.9429 |
| 43 | NAT | 0.0011 | 0.0069 | 0.9761 |
| 44 | 5HT1b | -0.0005 | -0.0188 | 0.9833 |
| Timepoint 4: C9NEG vs. HC (*N*=24 vs. *N*=24) | | | | |
| 1 | MU (map 2) | -0.0619 | -0.1423 | 0.9233 |
| 2 | VAChT | -0.065 | -0.1317 | 0.9233 |
| 3 | VAChT (map 3) | -0.0636 | -0.1125 | 0.9233 |
| 4 | SERT (map 3) | -0.0507 | -0.0985 | 0.9233 |
| 5 | MU | -0.0545 | -0.0935 | 0.9233 |
| 6 | VAChT (map 2) | -0.0623 | -0.0852 | 0.9233 |
| 7 | NMDA | -0.0353 | -0.0829 | 0.9233 |
| 8 | VMAT2 | -0.0447 | -0.0624 | 0.9233 |
| 9 | MOR | -0.0543 | -0.0601 | 0.9233 |
| 10 | MOR (map 2) | -0.057 | -0.0573 | 0.9233 |
| 11 | D1 | -0.0621 | -0.051 | 0.9233 |
| 12 | KappaOp | -0.0421 | -0.0495 | 0.9233 |
| 13 | FDOPA | -0.0443 | -0.0312 | 0.9233 |
| 14 | DAT | -0.0593 | -0.0284 | 0.9233 |
| 15 | SERT (map 2) | -0.0563 | -0.0232 | 0.9233 |
| 16 | 5HT4 | -0.0341 | -0.0219 | 0.9233 |
| 17 | SERT | -0.0627 | -0.015 | 0.9233 |
| 18 | A4B2 | -0.0229 | 0.0058 | 0.9233 |
| 19 | CB1 (map 2) | -0.0358 | 0.0124 | 0.9233 |
| 20 | Microglia | -0.0348 | 0.0154 | 0.9233 |
| 21 | GABAa5 (map 2) | 0.0326 | 0.0385 | 0.9233 |
| 22 | GABAa | -0.0426 | 0.0543 | 0.9489 |
| 23 | D2 (map 3) | -0.0246 | -0.034 | 0.9717 |
| 24 | NAT | 0.0131 | -0.003 | 0.9717 |
| 25 | 5HT1b | -0.0149 | 0.0067 | 0.9717 |
| 26 | 5HT1a (map 2) | -0.0286 | 0.0194 | 0.9717 |
| 27 | mGluR5 (map 2) | -0.0185 | 0.0205 | 0.9717 |
| 28 | KOR | -0.0225 | 0.0231 | 0.9717 |
| 29 | 5HT1a | -0.0278 | 0.0238 | 0.9717 |
| 30 | 5HT2a | 0.0196 | 0.0394 | 0.9717 |
| 31 | COX1 | -0.0027 | -0.0406 | 0.9748 |
| 32 | HDAC | -0.0027 | -0.0324 | 0.9748 |
| 33 | D2 (map 2) | -0.0048 | -0.025 | 0.9748 |
| 34 | 5HT1b (map 2) | -0.0041 | -0.0043 | 0.9748 |
| 35 | CMRglu | -0.0061 | 0.0016 | 0.9748 |
| 36 | mGluR5 (map 3) | -0.0033 | 0.0025 | 0.9748 |
| 37 | CB1 | 0.001 | 0.0094 | 0.9748 |
| 38 | CBF | -0.002 | 0.0213 | 0.9748 |
| 39 | 5HT2a (map 2) | 0.0072 | 0.0265 | 0.9748 |
| 40 | mGluR5 | 0.0108 | 0.0312 | 0.9748 |
| 41 | GABAa5 | 0.0163 | 0.0411 | 0.9748 |
| 42 | M1 | -0.0109 | 0.0457 | 0.9748 |
| 43 | 5HT6 | -0.0015 | 0.0529 | 0.9748 |
| 44 | SV2A | -0.0018 | 0.0679 | 0.9748 |

Neurotransmitters ranked based on the significance of their voxel-wise spatial correlations with atrophy patterns in specific group comparisons C9NEG vs. HC, C9POS vs. HC and C9POS vs. C9NEG at specific longitudinal timepoints. Results are ranked by p-value first (ascending), then by Median Spearman ρ (ascending). For more details and abbreviations of all neurotransmitter maps, please see the main manuscript.

Abbreviations: ALS: amyotrophic lateral sclerosis, C9POS: C9orf72-positive ALS, C9NEG: sporadic ALS without known ALS-associated genetic variants, HC: healthy controls, PET: positron emission tomography, p_FDR_BH: p-value adjusted for false discovery rate using the Benjamini-Hochberg procedure
